# Physics of emergence beyond Berezinskii–Kosterlitz–Thouless transition for interacting topological quantum matter

**DOI:** 10.1038/s41598-022-15834-y

**Published:** 2022-07-13

**Authors:** Ranjith R. Kumar, Sujit Sarkar

**Affiliations:** 1 Theoretical Sciences Division, Poornaprajna Institute of Scientific Research, Bidalur, Bengaluru, 562164 India; 2 Graduate Studies, Manipal Academy of Higher Education, Madhava Nagar, Manipal, 576104 India

**Keywords:** Topological matter, Phase transitions and critical phenomena

## Abstract

An attempt is made to find different emergent quantum phases for interacting topological state of quantum matter. Our study is based on the quantum field theoretical renormalization group (RG) calculations. The behaviour of the RG flow lines give the emergence of different quantum phases for non-interacting and interacting topological state of quantum matter. We show explicitly electron-electron interaction can turn a topologically trivial phase into a topologically nontrivial one and also topologically nontrivial phase to topologically trivial phase. We show that physics of emergence goes beyond the quantum Berezinskii–Kosterlitz–Thouless transition. We also present the analysis of fixed point and show the behaviour of fixed point changes in presence and absence of interaction. This work provides a new perspective not only from the topological state of interacting quantum matter and but also for the correlated quantum many -body physics.

## Introduction

The field of topological state of quantum matter is one of the most active research areas in experimental and also in theoretical quantum matter physics^1–7^. Topological materials are expected to be robust against disorder^8–13^. This makes topological materials candidates for new electronic devices. The physics of topological state of quantum matter is typically described by the band theory and symmetry of non-interacting fermions^1–7,9,10^. But in all solids, electron–electron interaction is unavoidable, either screened or weak or sometimes even stronger dominate the physical properties of a material^9,10,14^ electron-electron interaction leads to the different physical phenomena in quantum many-body systems, such as the Kondo effect, Mott-Hubbard transition and superconductivity to mention a few^9,10,14^. Therefore, to get a complete picture of the topological state of a quantum many-body system, one has to consider the effect of interaction^15–32^.

Models of strongly correlated electrons have provided outstanding challenges to condensed matter theory for many decades^10,14^. Most of the previous work on topological superconductivity in one-dimensional wires has focused on for the noninteracting limit^1–7,11^. Interaction induced topological phases such as topological Kondo insulator, topological Mott insulator and fractional Chern insulator only exists due to the interplay of topology and strong correlations^11^.

We expect the presence of interaction in the physical system makes it much more complex than the simple non-interacting topological state of quantum matter^15–32^.

The physics of one dimensional quantum many body is interesting in its own right^33,34^. One dimensional quantum many body systems have strong quantum fluctuations that do not allow spontaneously broken continuous symmetries. As a result of this, the pairing instabilities do not lead to any ordered density-wave^10^. This many body has a critical phase with power law decay of various correlation functions, universally known as a Luttinger liquid^10,14^. In one dimensional quantum many body systems whether it is weakly correlated or strongly proper treatment of the quantum fluctuations leads in both cases to a Luttinger liquid characterized by phonon-like collective density fluctuation modes.

In the present study, we use the bosonization process to recast the model Hamiltonian in continuum field theory. Our systems can be mapped to a dual-field double sine-Gordon model as a bosonized effective field theory. The mathematical structure and results of the RG theory are a significant conceptual advancement in the quantum field theory of both high-energy and condensed matter physics^35–37^ in the last several decades.

## Motivation

Emergent phenomenas are essential aspects^10^ in quantum many body physics. In this view, fundamentally new kinds of phenomena emerge in the different region of parameter space of quantum many body system. In this paper, we study various quantum emergence phases for an one-dimensional one-component fermions having proximity-induced pairing gap and inter particle short-range interaction, as a generalization of the Kitaev model. In this study we raise the question and also solve how electron-electron interaction turns a non-trivial topological state to a topological trivial state and also a topological non-trivial state to topological trivial state for interacting topological quantum matter.

The other motivation of this study is to do the detailed analysis of fixed points of RG theory for the non-interacting and interacting topological states of our model Hamiltonian system. It leads us to many interesting features of stable and unstable phases of our model Hamiltonian systems. The most important part of this study is to show that the emergent quantum phases are far richer and go beyond the Berizinskii-Kosterlitz-Thouless (BKT) transition^38,43^.

**Figure 1:**
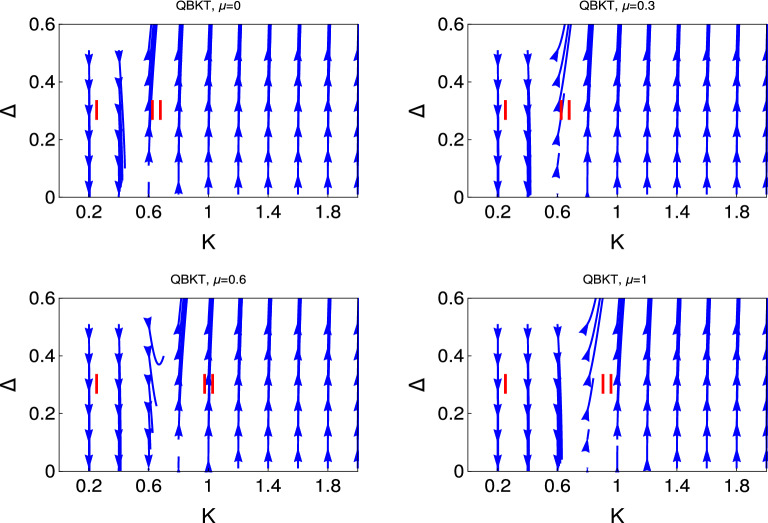
Behaviour of the RG flow lines in the $\Delta$-*K* plane. We present the RG flow lines based on the solution of non-interacting RG equations (Eq. 3).

**Figure 2:**
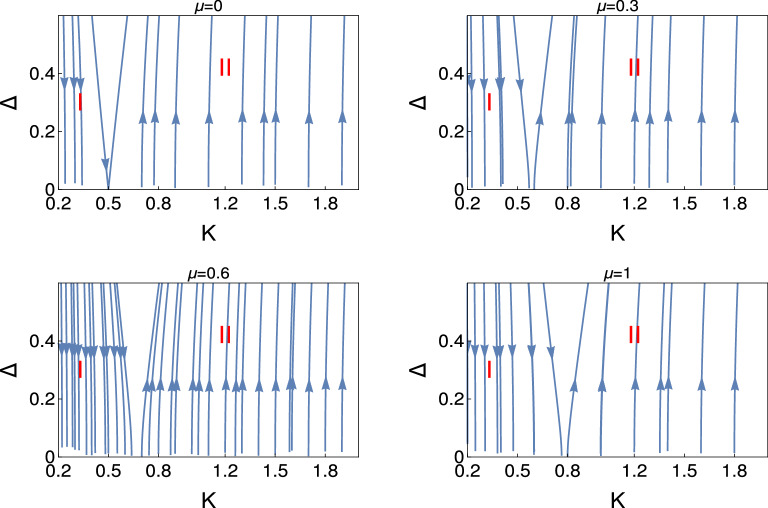
Behaviour of the RG flow lines in the $\Delta$-*K* plane. We present the RG flow lines based on the exact solution (Eq. 4).

### Model hamiltonians and renormalization group equations for non-interacting ($U=0$) quantum matter

We consider the non-interacting Kitaev chain^44,45^ as the model Hamiltonian for the non-interacting topological state of quantum matter,

1 $$\begin{aligned} H_1 = - t \sum _{i=1}^{N-1} ({c_i}^{\dagger } {c_{i+1}} + h.c ) + \Delta \sum _{i=1}^{N-1} ( {c_i} {c_{i+1}} + h.c ) -{\mu } \sum _{i}^{N} {c_i}^{\dagger } {c_i} . \end{aligned}$$

*t* is the hopping integral for nearest-neighbour sites, $\Delta$ is the proximity induced superconducting gap and $\mu$ is the chemical potential.

Bosonized version of the model Hamiltonian is the following,

2 $$\begin{aligned} H = H_0 + \frac{\Delta }{2} \int \cos \left( 2 \sqrt{\frac{\pi }{K}}\theta (x)\right) dx + \mu \sqrt{\frac{K}{\pi }} \int (\partial _x \phi (x)) dx, \end{aligned}$$

where $H_0 = \frac{v}{2} \int [(\partial _x \theta )^2+ (\partial _x \phi )^2] dx$ is the non-interacting part of the Hamiltonian and *v* is the collective velocity of the system and *K* is the Luttinger liquid parameter of the system^14^ (detail derivation is relegated to the “Method” section, where we present how “K” appears in the bosonized form of the model Hamiltonian).

We express our model Hamiltonian in terms of two dual fields $\theta (x)$ and $\phi (x)$, which bosonized the Hamiltonian. The fermionic fields for right (*R*) and left (*L*) movers of a one dimensional quantum many body system are ${\psi }_{R/L, \uparrow / \downarrow } (x) = \frac{1}{2 \pi \alpha } {\eta _{R, \uparrow }}~ e^{i \sqrt{4 \pi } {\phi }_{R, \uparrow / \downarrow } (x) }$, where $\eta _{L/R}$ is the Klein factor to preserve the anticommutivity of the fermionic field which obeys Clifford algebra^14^. These two fields are related by the relations, $\phi (x) = {\phi }_R (x) + {\phi }_L (x)$ and $\theta (x) = {\theta }_R (x) + {\theta }_L (x)$.

The analytical expressions for the non-interacting RG equations are the following (detailed derivation is relegated to the “Method” section),

3 $$\begin{aligned} \frac{d \Delta }{dl}&=\left[ 2-\frac{\alpha }{K}\right] \Delta \nonumber \\ \frac{dK}{dl}&= \frac{{\Delta }^2}{8} . \end{aligned}$$

$$ \alpha = \left( 1+ \frac{\mu }{\pi \sqrt{\pi }} \right) $$, ( $$\alpha =1 $$, for $$\mu = 0 $$).

Exact solution for $\Delta$ (detail derivation is relegated to the “Method” section)

4 $$\begin{aligned} \Delta = \sqrt{ {{\Delta }_0}^2 + 32 (K - K_0 ) -16 ln(K/K_{0} ) ( 1 + \frac{\mu }{\pi \sqrt{\pi }} )}. \end{aligned}$$

### Model Hamiltonians and renormalization group equations for interacting ($U \ne 0$) quantum matter

The model Hamiltonian for the interacting Kitaev model is,

5 $$\begin{aligned} H_2 = - t \sum _{i=1}^{N-1} ({c_i}^{\dagger } {c_{i+1}} + h.c ) + \Delta \sum _{i=1}^{N-1} ( {c_i} {c_{i+1}} + h.c ) + U \sum _{i=1}^{N-1} ( 2 {c_i}^{\dagger } {c_i} - 1) ( 2 {c_{i+1}}^{\dagger } {c_{i+1}} - 1) -{\mu } \sum _{i}^{N} {c_i}^{\dagger } {c_i} . \end{aligned}$$

The bosonized form of the interacting Kitaev model Hamiltonian is,

6 $$\begin{aligned} H_2 = H_0 + \frac{\Delta }{2} \int \cos \left( 2 \sqrt{\frac{\pi }{K}}\theta (x)\right) dx + U \int \cos \left( 4 \sqrt{\pi K}\phi (x)\right) dx - \mu \sqrt{\frac{K}{\pi }} \int (\partial _x \phi (x)) dx, \end{aligned}$$

the third term (*U*) represents the intersite repulsive interaction. In this model Hamiltonian, there is no on-site repulsion owing to the Pauli exclusion principle.

It is very clear from the above Hamiltonian that our model Hamiltonian contains two strongly relevant and mutually nonlocal perturbations over the critical theory. For the present Hamiltonian, the strong coupling fixed point is usually determined by the most relevant perturbation whose amplitude grows up according to its Gaussian scaling dimensions and it is not much affected by the less relevant coupling terms. But, this is not the general rule if the two operators exclude each other. For the above Hamiltonian, the interplay between the *U* and $\Delta$ relevant operators which are related with dual fields $\phi (x)$ and $\theta (x)$ can produce a novel quantum phase transition through a critical point or a critical line. Thus we consider RG study to solve the interacting and non-interacting topological state of quantum matter. For this reason, this work provides a new perspective for the topological state of interacting quantum matter and also for the correlated quantum many body physics. We derive the RG equations in the perturbative regime, therefore during the studies of RG flow diagram, we consider the smaller initial values of the couplings. The analytical expressions for the interacting RG equations are the (detailed derivation is relegated to the “Method” section),

7 $$\begin{aligned} \frac{d \Delta }{dl}&= \left[ 2-\frac{\alpha }{K} \right] \Delta + 2 K \Delta U^2 \nonumber \\ \frac{d U}{dl}&= \left( 2-4K \right) U \nonumber \\ \frac{dK}{dl}&= \frac{{\Delta }^2}{8} - 8 K^2 U^2 . \end{aligned}$$

$$ \alpha = \left( 1+ \frac{\mu }{\pi \sqrt{\pi }} \right) $$, ( $$\alpha =1 $$, for $$\mu = 0 $$).

## Results

### Emergence of quantum phases for non-interacting topological state of quantum matter

We have derived the RG equations in the perturbative limit, therefore we consider small values initial values of the couplings during the study of RG flow diagrams.

In Fig. 1, we present the behaviour of RG flow lines in $\Delta$ -*K* plane from the solution of RG equations (Eq. 3). We study the RG flow lines behaviour of couplings with *K* for the following reasons: The physics of low-dimensional quantum many-body condensed matter system is enriched with its new and interesting emergent behavior with the parameter *K*^14^. In one dimensional quantum many-body system, *K*, plays an important role to determine the different emergent quantum phases. $K < 1$, $K > 1$ and $K=1$ are respectively charactering the repulsive, attractive and non-interacting^14^ state of the system. We present four figures for different values of $\mu$. It reveals from the behaviour of RG flow lines that the upper panel, the left and right figures are respectively for $\mu =0$ and $\mu =0.3$. It has only two quantum phases one is the weak coupling phase, (I), and the other is the strong coupling phase (II). There is no evidence of phase crossover from weak coupling to strong coupling or vice versa. In the lower panel, the left and right figures are respectively for $\mu =0.6$ and $\mu =1$. The qualitative behaviour of the RG flow lines are the same but the gapless LL phase region increase as we increase $\mu$.

In Fig. 2, we present the exact solution for the RG flow equation for the coupling $\Delta$ based on Eq. (4). This figure panels consists four figures for different values of $\mu$ as depicted in the figures. It is very clear from these figures that for this RG flow diagram system is in always in two phase regime, i.e., either in the weak coupling phase or in the strong coupling phase. It reveals from this study that for $\mu =0$ this transition occurs for $K=0.5$. As we increase the value of $\mu$ the transition points shift from $K =0.5$. This transition point shifts to the higher value of *K* for the higher values of $\mu$. This exact solution study is consistent with the numerical study of the RG flow equations in Fig. 1.

### Emergence of quantum phases for interacting topological state of quantum matter

Now we present the result of interacting Kitaev model to show the emergence of different quantum phases in presence of electron-electron interaction.

**Figure 3:**
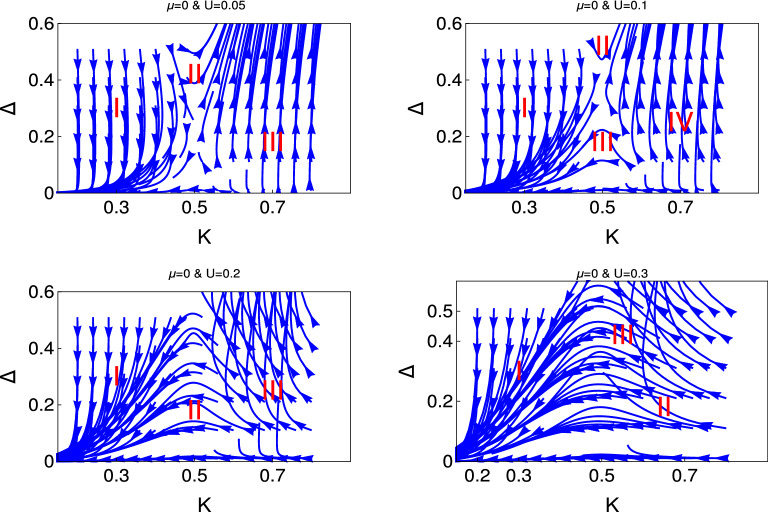
Behaviour of the RG flow lines in the $\Delta$- *K* plane for the different initial values of $U(0) = (0.05, 0.1, 0.2, 0.3)$. Here we consider $\mu =0$. We present the RG flow lines from the study of RG equation (Eq. 7).

**Figure 4:**
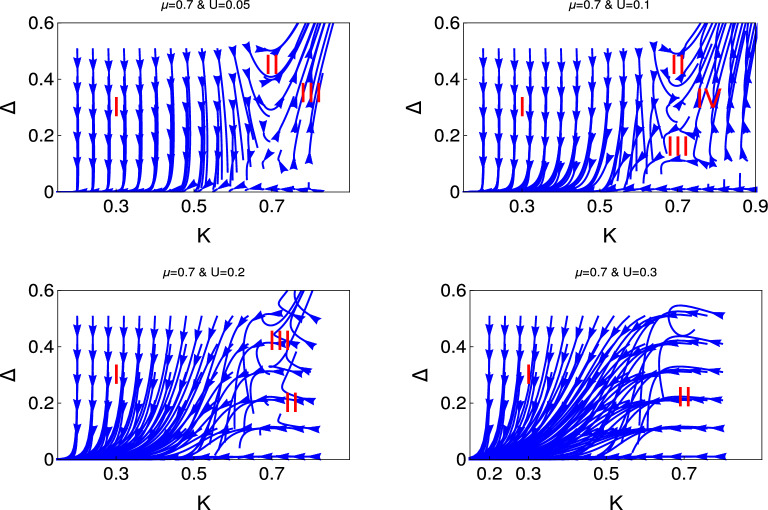
Behaviour of the RG flow lines in the $\Delta$- *K* plane for the different initial values of $U(0) = (0.05, 0.1, 0.2, 0.3)$. Here we consider $\mu =1$. We present the RG flow lines from the study of RG equation (Eq. 7).

Figure 3 shows the behaviour of RG flow lines (Eq. 7) for the coupling $\Delta$ for the different initial values of *K*. This figure panel consists of four figures for the different initial values of *U* as depicted in the figures and for $\mu =0$. It reveals from the behaviour of the RG flow lines of the left upper panel, it consists of three different phase regions. The system is in the weak coupling phase in region (I), where the RG flow lines are flowing off to the weak coupling phase and finally touch the base line. Region (II) is the phase crossover region from weak coupling phase to the strong coupling phase, i.e., finally systems drives to the gapped phase, we term this phase crossover as first phase crossover. In region (III), RG flow lines are flowing off to the strong coupling phase.

**Figure 5:**
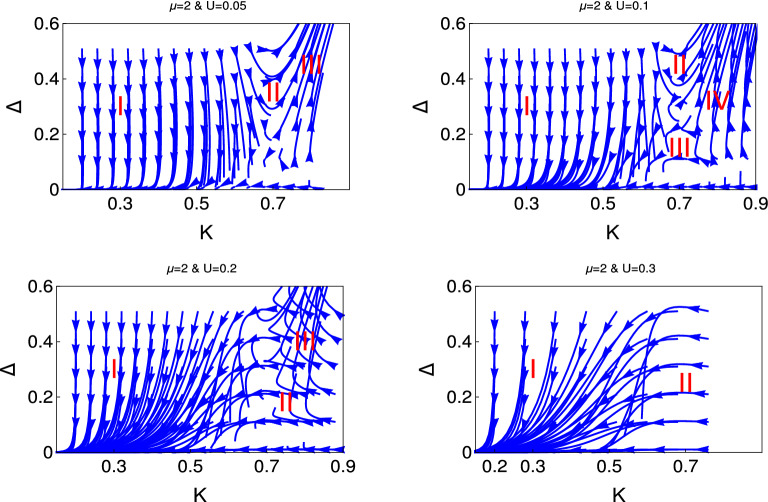
Behaviour of the RG flow lines in the $\Delta$- *K* plane for the different initial values of $U(0) = (0.05, 0.1, 0.2, 0.3)$. Here we consider $\mu =0$. We present the RG flow lines from the study of RG equation (Eq. 7).

The right figure of the upper panel is for the higher initial values of $$U (=0.1) $$, the most interesting feature of this RG flow diagram is the appearance of extra phase crossover from strong coupling to the weak coupling due to the reverse flow of the RG flow lines. We term this phase crossover as second phase crossover (III) and region (IV) is the strong coupling phase.

It reveals from the further higher initial values of $U (=0.2 )$ that the second crossover phase take the dominant region and the first crossover region totally disappear. We observe the appearance strong coupling phase for higher initial values of *K* and $\Delta$.

The most interesting feature that we observe for further initial values of $$U =0.3$$. We observe weak coupling phase in region I. For the higher initial values of $$\Delta (\le 0.22)$$, the initial phase of the RG flow lines are strong coupling phase but the direction of these RG flow lines change to the weak coupling phase around $$ K =0.5$$ and sharply touches the base line.

Another interesting phase we observe from this study is the appearance of a different type of phase crossover. We mark this region of phase crossover as region (II). In this phase crossover region system drives from flat phase to the weak coupling phase. We term this phase crossover as a third phase crossover. This flat phase occurs for the higher initial values of *K* ($\sim 0.8$) and for the smaller initial values of $\Delta (\sim 0.2)$. These RG flow lines are flowing off with constant values and finally reach the weak coupling phase and touch the base line.

**Figure 6:**
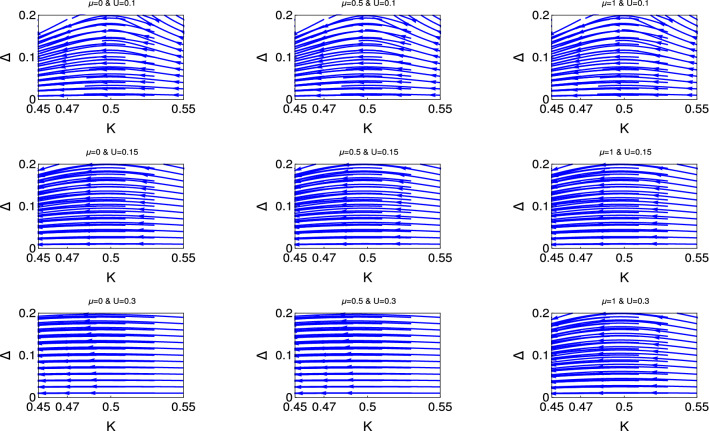
Behaviour of the RG flow lines for the couplings in the $\Delta$-*K* plane. We present the RG flow lines based on the solution of Eq. 7.

In Figs. 4 and 5, we present the results for $\mu = 0.7$ and 2 respectively for the same RG equation for interacting Kitaev chain. The behaviour of RG flow lines for the higher values of $\mu$ are differ from the $\mu =0$ in the following manners.

1. For $U= 0.05$, the phase crossover region shifted to the higher values of *K*.
2. For $U =0.1$, the second phase crossover region is very faint for $\mu =0.7$ and finally almost disappear for $\mu =2$.
3. Flat phase appears for the smaller initial value of $U =0.2$.
4. For higher initial values of $U=0.3$, we predict only two phases and there is no evidence of first phase crossover region, as we observe for $\mu =0$. We observe weak coupling phase and phase crossover from the flat phase region.

Thus it is clear from this study how the chemical potential drives to the different emergent phases for same value of electron-electron interaction.

Fig. 6 consists of three panels. The upper, middle and lower are respectively for $U = 0.1, 0.15$ and 0.3. Each panel consists of three figures for three different values of $\mu$, the left, middle and right figures are respectively for $\mu =0 , 0.5$ and 1. The main emphasis of this study is to show explicitly the transition from the second phase crossover to the flat phase region. The upper panel shows the evidence of second phase crossover for all values of chemical potential. We observe that for the higher initial values of *U*, as we notice in the second and third panel. The second phase crossover has started slowly and finally appears as a flat phase for higher values of $U (=0.3)$. Thus we show explicitly how the electron-electron interaction turns the topologically non-trivial state to topologically trivial phase.

#### Physical interpretation of quantum emergence phases and phase crossovers for non-interacting and interacting topological quantum states

We present the results for non-interacting and interacting topological state of quantum matter through Figs. 1, 2, 3, 4, 5, and 6. We observe the emergence of three different quantum phases and three phase crossovers regions. Now we physically interpret these quantum phases and phase crossover regions. Weak coupling phase for the non-interacting system is the gapless Luttinger liquid phase and the strong coupling phase is the proximity induce topological superconductng phase (Figs. 1 and 2).

For the interacting system, this weak coupling phase is the charge density wave phase (CDW) phase due to the electron-electron interaction and the strong coupling phase is the proximity induce topological superconducting phase. The first phase crossover region is the phase crossover of the system from CDW phase to the topological superconducting phase. The second phase crossover region is the phase crossover from topological superconducting phase to the CDW phase. There is a possibility of unstable equilibrium state between these two crossover regions. It is neither the CDW phase nor the proximity induced topological superconducting phase. Flat phase is for the constant initial value of topological superconducting phase and it is always associated with the third phase crossover to the weak coupling CDW phase. There is no evidence of phase crossover from flat phase to strong coupling phase. In the third phase cover regions the initial topological superconducting phase is constant over a large region of *K*. Whereas for the second phase crossover regions the initial topological superconducting phase has started to decreases from the very beginning.

It reveals from our study that there are only two phase regions for non-interacting phase, there are no phase crossover regions and the topological superconducting regions shifted with the chemical potential. We have observed that for interacting phase, three different kind of phase crossover regime have appeared as a function of electron-electron interaction and chemical potential. Thus the electron-electron interaction can turn topologically trivial phase to the topologically non-trivial phase and also the topologically non-trivial phase to the topologically trivial phase. We have observed that for the higher values of chemical potential and also for the higher values of *U*, there is no evidence of proximity induced topological superconducting phase. To the best of our knowledge this is the first study in the literature where the effect of electron-electron interaction and the effect of chemical potential has studied for topological state of quantum matter rigorously along with the physical interpretation.

### Characterization of fixed points and stability analysis

Now we present the nature of fixed points and stability analysis^46,47^ for the non-interacting and interacting Hamiltonians.

Stable fixed points: The scaling fields are become irrelevant. These fixed points are for the stable phase for matter. This fixed point behave as a attractor. When one releases the system in the parameter space close to these fixed points , it scales towards to this fixed point and eventually sits there. These fixed point is impervious to moderate variations in the microscopic morphology of the system.

Unstable fixed points: The scaling fields are become relevant. These fixed points are not for the stable phase for matter. The quantum phase of matter in this fixed point will not be stable phase of matter, if the RG flow lines approach to this fixed point, finally it will be away from it.

Marginal fixed points: Marginal scaling field corresponds to a direction in coupling constant space with vanishing partial derivative. For this situation one can consider the second order derivative ${{\partial }^2 R}|_{g^{\star =0 }}= 2 x$. In the vicinity of this fixed point, the scaling field then behaves as $d v_{\alpha } = x {v_{\alpha }}^2$. The scaling field is marginally relevant and irrelevant for $x >0$ and $x < 0$ respectively.

#### Stability matrix for the non-interacting RG equations (Eq. 3)

Now we do the stability analysis for the fixed point analysis of RG equation (3) (detail derivation is relegated to the “Method” section).

$$\frac{d A_1 }{dl} = B_1 A_1 $$, $$A_1 = {( \delta K, \delta \Delta )}^{T} $$

8 $$\begin{aligned} B_1 = \left( \begin{matrix} 0 &{}&{} \frac{{\Delta }^{\star }}{4} \\ \frac{\alpha {\Delta }^{\star }}{ {K^{\star }}^2 } &{}&{} (2 - \frac{\alpha }{ K^{\star } }) \\ \end{matrix}\right) , \end{aligned}$$

#### Stability matrix for the interacting RG equation (Eq. 7)

$$\frac{d A_2 }{dl} = B_2 A_2 $$, $$A_2 = {( \delta K , \delta U , \delta \Delta )}^{T} $$

9 $$\begin{aligned} B_2 = \left( \begin{matrix} -16 {{U}^{\star }}^2 K^{\star }&{}&{} -8 {{U}^{\star }} {K^{\star }}^2 &{}&{} \frac{{\Delta }^{\star }}{4}\\ - 4 {U}^{\star } &{}&{} (2 -4 K) &{}&{} 0 \\ C &{}&{} 4 {K}^{\star } {\Delta }^{\star } {U}^{\star } &{}&{} D \\ \end{matrix}\right) , \end{aligned}$$

where $C = \frac{\alpha }{{ K^{\star }}^2} + 2 {\Delta }^{\star } {{U}^{\star }}^{2}$, $D = (2 - \frac{\alpha }{ K^{\star }}) + 2 {K}^{\star } {{U}^{\star }}^{2}$, and ${U}^{\star }$, ${\Delta }^{\star }$ and ${K}^{\star }$ are the value of *U*, $\Delta$ and *K* at the fixed point.

## Results from the analysis of the fixed point

### Stability of phase analysis for non-interacting systems

The detail derivation of the analysis of fixed points are relegated in the “Method” section.

**Table 1:** Results of the fixed point analysis for the non-interacting RG equation (Eq. 3) for $\Delta$.

K	1/2	1	1.5
$\alpha =1$	One relevant and one marginal	One relevant and one marginal	All are marginal
$\alpha =1.5$	One irrelevant and one marginal coupling	One irrelevant and one marginal	One relevant and one marginal
$\alpha =2$	One relevant and one marginal	All are marginal	One relevant and one marginal

**Table 2:** Results of the fixed point analysis for the interacting RG equation (Eq. 7).

K	1/2	1	1.5
$\alpha =1$	All are marginal	One irrelevant and two marginal	One relevant, one irrelevant and one marginal
$\alpha =1.5$	One irrelevant and two marginal	One relevant, one irrelevant and one marginal	One relevant, one irrelevant and one marginal
$\alpha =2$	One irrelevant and two marginal	One irrelevant and two marginal	One relevant, one irrelevant and one marginal

The analysis of table-I is for the non-interacting RG equation. This table consists of three rows and three column. Each row is for the different values of $\alpha$ and each column is for the different values of *K*.

It is clear from the non-interacting case for zero chemical potential ($$ \alpha =1$$ ), there is no stable phase. But for finite chemical potential ($$\alpha = 1.5$$ ), the system has stable phase for $$K=1/2 $$ and $$K=1 $$ regime but for $$ K= 1.5$$ regime. The system has again no stable system. We find for further higher values of chemical potential ($$\alpha = 2 $$) system has no stable phase.

### Stability of phase analysis for interacting systems

The analysis of table-II is for the interacting RG equation. This table consists of three rows and three column. Each row is for the different values of $\alpha$ and each column is for the different values of *K*.

It is clear from the interacting case for zero chemical potential ($\alpha =1$ ), there is stable phase exists only for $K=1$ and $K =1.5$. But for finite chemical potential ($\alpha = 1.5$ ), the system has stable phase for the all values of *K*. We find for further higher values of chemical potential ($\alpha = 2$) system has also in the stable phase for all regime.

#### Physics of emergence beyond the quantum Berezinskii-Kosterlitz- Thouless transition

The BKT mechanism^38–43^, in which a phase transition is mediated by the proliferation of topological defects, governs the critical behavior of a wide range of equilibrium two dimensional systems with a continuous symmetry, ranging from spin systems to superconducting thin films and two-dimensional Bose fluids, such as liquid helium and ultracold atoms^38–43^.

The physics of emergence of quantum phases in low dimensional quantum many body system is an essential phenomena. Many of the systems have shown the appearance of quantum emergence phases and their behaviour is alike to the QBKT transition^14,32,47^. We now discuss the results of emergence quantum physics which we have obtained in this study.

The physics of QBKT possesses two phase regions, weak coupling and strong coupling, and a single phase crossover region. In weak coupling, system is gapless, and in the strong coupling phase system is in the gapped phase and the coupling is in the relevant phase in the sense of RG. The phase crossover region is associated with the phase crossover from weak coupling to the strong coupling phase.

We observe the two phases region from the study of the behaviour of RG flow lines for non-interacting topological quantum matter, one is the weak coupling phase and the other is the strong coupling phase.

For the interacting topological state of matter, we observe the two phase regions and one phase crossover region for the lower values of electron-electron interaction which is consistent with the QBKT physics. We have observed emergence of different quantum phases and phase crossovers, as we increase the value of $\mu$ and *U*. We observe two phases region for both non-interacting and interacting topological state of quantum matter but the characters are different for non-interacting and interacting topological state of quantum matter. Thus the emergent physics of topological excitation for interacting quantum matter is far more enriched than the conventional QBKT.

## Discussions

We have shown that there is no phase crossover physics for non-interacting topological state of quantum matter, this system has only two quantum emergence phases. We have shown explicitly that there are three phase crossovers regions and also three quantum phases for interacting topological state of quantum matter. We have shown explicitly that how the electron-electron interaction can turn a topologically trivial state to topologically non-trivial state at the same time we have also shown how the electron-electron interaction can turn a nontrivial topological phase to topologically trivial phase. We have shown the physics of emergence for interacting topological state of quantum matter is beyond the BKT transition. This work provides a new perspective for the topological state of interacting quantum matter and also for the correlated quantum many body physics.

## Method

### Scaling law and critical exponent

It is well known in the literature that the diverging coherence length ($\xi \rightarrow \infty$) is the signature of second order quantum phase transition. Here we discuss the basic physics and mathematical analysis of the fixed points and stability analysis of the RG equations and from that analysis we interfere about the stable and unstable phases of the system^46,47^.

Our main interest is to study the behaviour of the flow in the immediate vicinity of the fixed point manifolds. One can do the stability analysis from the following condition. When the coupling constant $\lambda$ is close to the fixed point ${\lambda }^*$.

$$ R( \lambda ) \equiv R (\lambda - {\lambda }^* + {\lambda }^* ) \simeq W (\lambda -{\lambda }^* ) $$, where $$W_{ab} = {( \frac{\partial R_a}{\partial g_b } )|}_{\lambda = {\lambda }^* } $$.

To explain the properties of RG flow lines and also the nature of fixed point, we consider the following steps. At first, we diagonalize the matrix *W* , suppose our coupling parameter space is *W* then the Eigenvalues are $g_1 , g_2 , .........g_N$. The left eigenvector is ${\phi }^{\alpha }$ then the eigen value equation ${{\phi }_{\alpha } }^T W = {{\phi }_{\alpha }}^{T} {\lambda }_{\alpha }$.

Let $v_{\alpha }$ be the $\alpha$th component of the vector, $\lambda - {\lambda }^*$ when represented in the basis $\{ \phi _{\alpha } \}$, we can write $v_{\alpha } = { {\phi }_{\alpha }}^{T} (\lambda - {\lambda }^{*} )$. These concept display a particularly simple behaviour under renormalization.

10 $$\begin{aligned} \frac{d v_{\alpha } }{d l } = {\phi _{\alpha }}^{T} \frac{d (\lambda - {\lambda }^* )}{d l } = {\phi _{\alpha }}^{T} W (\lambda - {\lambda }^* ) = {\lambda }_{\alpha } v_{\alpha } , \end{aligned}$$

where $v_{\alpha }$ is the scaling field which changes by the scaling factor thus $v_{\alpha } \simeq e^{l \lambda _{\alpha } }$. For ${\lambda }_{\alpha } > 0$, the flow is directed away from the critical point and this scaling field is the relevant one. When $\lambda _{\alpha } < 0$, the flow is attracted by the fixed point and the scaling field is the irrelevant one. The scaling field which are invariant under the flow, ${\lambda }_{\alpha } =0$ are

### Analysis of fixed point for the non-interacting RG equations (Eq. 3)

For this case, we consider the three different fixed points for all values of $\alpha$. These three different fixed points correspond to the different correlated phases of the system.

1. Here we consider $\alpha = 1 (\mu = 0)$ ($K^{\star }$, ${\Delta }^{\star }$) = (1/2, 0) : Eigenvalues and eigenfunctions of the stability matrix analysis are the following: Eigenvalues are 1, 0, corresponding eigenfunctions are (0, 1), (1, 0). ($K^{\star }$, ${\Delta }^{\star }$) = (1, 0) : Eigenvalues and eigenfunctions of the stability matrix analysis are the following: Eigenvalues are 1.333, 0, corresponding eigenfunctions are (0, 1), (1, 0). ($K^{\star }$, ${\Delta }^{\star }$) = (1.5, 0) : Eigenvalues and eigenfunctions of the stability matrix analysis are the following: Eigenvalues are 0, 0, corresponding eigenfunctions are (0, 1), $(-1, 0)$. We consider $\alpha > 1$ ( i.e., $\mu \ne 0$, ${\Delta }^{\star } =0$ ). For this case, we consider the three different fixed points.
2. Here we consider $$\alpha = 1.5 $$ ($$ K^{\star }$$, $${\Delta }^{\star }$$) = (1/2, 0) : Eigenvalues and eigenfunctions of the stability matrix analysis are the following: Eigenvalues are $$-1, 0 $$, corresponding eigenfunctions are (0, 1), $$(-1, 0)$$. ($$ K^{\star }$$, $${\Delta }^{\star }$$) = (1, 0) : Eigenvalues and eigenfunctions of the stability matrix analysis are the following: Eigenvalues are 0.5, 0, corresponding eigenfunctions are (0, 1), $$(-1, 0)$$. ($$ K^{\star }$$, $${\Delta }^{\star }$$) = (1.5, 0) : Eigenvalues and eigenfunctions of the stability matrix analysis are the following: Eigenvalues are 1, 0, corresponding eigenfunctions are (0, 1), $$(-1, 0)$$.
3. Here we consider $$\alpha = 2 $$ ($$ K^{\star }$$, $${\Delta }^{\star }$$) = (1/2, 0) : Eigenvalues and eigenfunctions of the stability matrix analysis are the following: Eigenvalues are $$-2, 0 $$, corresponding eigenfunctions are (0, 1), (1, 0). ($$ K^{\star }$$, $${\Delta }^{\star }$$) = (1, 0) : Eigenvalues and eigenfunctions of the stability matrix analysis are the following: Eigenvalues are 0, 0, corresponding eigenfunctions are (0, 1), (1, 0). ($$ K^{\star }$$, $${\Delta }^{\star }$$) = (1.5, 0) : Eigenvalues and eigenfunctions of the stability matrix analysis are the following: Eigenvalues are 0.667, 0, corresponding eigenfunctions are (0, 1), $$(-1, 0)$$.

### Stability analysis for the fixed point of interacting topological RG equations (Eq. 7)

Now we do the fixed point analysis for the whole set of RG equation. Here we take three different values of *K*, which correspond to the three different region of interaction, i.e, strongly correlated regime ($$K=0.5$$), non-interacting regime ($$K=1$$) and attractive regime ($$K=1.5 $$). We also present the results three different values of $$\mu $$ for each *K*.

1. At first we consider $\alpha = 1$ ($\mu =0$, $U^{\star } =0$, ${\Delta }^{\star } =0$). For this case, we consider the three different fixed points. ($K^{\star }$, $U^{\star }$, ${\Delta }^{\star }$) = (1/2, 0, 0) : Eigenvalues and eigenfunctions of the stability matrix analysis are the following: Eigenvalues are 0, 0, 0, corresponding eigenfunctions are (1, 0, 0), (0, 1, 0), (0, 0, 1). Here all the couplings are the marginal, there is no evidence of a stable phase. ( $K^{\star }$, $U^{\star }$, ${\Delta }^{\star }$) = (1, 0, 0) : Eigenvalues and eigenfunctions of the stability matrix analysis are the following: Eigenvalues are $-2, 1, 0$, corresponding eigenfunctions are (0, 1, 0), (0, 0, 1), (1, 0, 0). Here we notice that one of the coupling is irrelevant which corresponds to the stable phase of the system. The other two couplings are the relevant and marginal. The relevant coupling corresponds to the unstable phase and the marginal coupling behave as irrelevant coupling when its approach to the fixed point but but it behave as relevant coupling when it very close or slightly away from the fixed point as we have shown through mathematical analysis. Marginal coupling never gives the stable fixed point. ( $K^{\star }$, $U^{\star }$, ${\Delta }^{\star }$) = (1.5, 0, 0) : Eigenvalues and eigenfunctions of the stability matrix analysis are the following: Eigenvalues are $-4, 1.33, 0$, corresponding eigenfunctions are (0, 1, 0), (0, 0, 1), (1, 0, 0). Here we notice that one of the coupling is irrelevant which corresponds to the stable phase of the system. The other two couplings are the relevant and marginal. The relevant coupling corresponds to the unstable phase. There is no evidence stable phase for this coupling. We consider $\alpha > 1$ ( i.e., $\mu \ne 0$, $U^{\star } =0$, ${\Delta }^{\star } =0$ ). For this case, we consider the three different fixed points, which corresponds to the different correlated regions.
2. Here we consider $\alpha =1.5$, ($K^{\star }$, $U^{\star }$, ${\Delta }^{\star }$) = (1/2, 0, 0) : Eigenvalues and eigenfunctions of the stability matrix analysis are the following: Eigenvalues are $-1, 0, 0$, corresponding eigenfunctions are (0, 0, 1), (0, 1, 0), (1, 0, 0). Here we notice that one of the coupling is irrelevant which corresponds to the stable phase of the system. The other two couplings are the marginal. The marginal coupling corresponds to the unstable phase. There is no evidence critical surface.
  (E). ($K^{\star }$, $U^{\star }$, ${\Delta }^{\star }$) = (1, 0, 0) : Eigenvalues and eigenfunctions of the stability matrix analysis are the following: Eigenvalues are $-2, 0.5, 0$, corresponding eigenfunctions are (0, 1, 0), (0, 0, 1), (1, 0, 0). Here we notice that one of the coupling is irrelevant which corresponds to the stable phase of the system. The another two couplings are the relevant and marginal. The relevant coupling corresponds to the unstable phase. There is no evidence critical surface.
  (F). ($K^{\star }$, $U^{\star }$, ${\Delta }^{\star }$) = (1.5, 0, 0) : Eigenvalues and eigenfunctions of the stability matrix analysis are the following: Eigenvalues are $-4, 1, 0$, corresponding eigenfunctions are (0, 1, 0), (0, 0, 1), (1, 0, 0). Here we notice that one of the coupling is irrelevant which corresponds to the stable phase of the system. The other two couplings are the relevant and marginal. The marginal coupling corresponds to the unstable phase. There is no evidence critical surface. Here we consider $\alpha = 2$,
  (G). ($K^{\star }$, $U^{\star }$, ${\Delta }^{\star }$) = (1/2, 0, 0) : Eigenvalues and eigenfunctions of the stability matrix analysis are the following: Eigenvalues are $-2, 0, 0$, corresponding eigenfunctions are (0, 0, 1), (0, 1, 0), (1, 0, 0). Here we notice that one of the coupling is irrelevant which corresponds to the stable phase of the system. The other two couplings are the marginal couplings. The marginal coupling corresponds to the unstable phase. There is no evidence critical surface.
  (H). ($K^{\star }$, $U^{\star }$, ${\Delta }^{\star }$) = (1, 0, 0) : Eigenvalues and eigenfunctions of the stability matrix analysis are the following: Eigenvalues are $-2, 0, 0$, corresponding eigenfunctions are (0, 1, 0), (1, 0, 0), (0, 0, 1). Here we notice that one of the coupling is irrelevant which corresponds to the stable phase of the system. The other two couplings are the marginal couplings. The marginal coupling corresponds to the unstable phase. There is no evidence critical surface.
  (I). ($K^{\star }$, $U^{\star }$, ${\Delta }^{\star }$) = (1.5, 0, 0) : Eigenvalues and eigenfunctions of the stability matrix analysis are the following: Eigenvalues are $-4, 0.66, 0$, corresponding eigenfunctions are (0, 1, 0), (0, 0, 1), (1, 0, 0).

### Derivation of bosonized Hamiltonian

11 $$\begin{aligned} H_{1}&= - t \sum _{i=1}^{N-1} ( {c_i}^{\dagger } {c_{i+1}} + h.c ) + \Delta \sum _{i=1}^{N-1} ( {c_i} {c_{i+1}} + h.c ) -{\mu } \sum _{i}^{N} {c_i}^{\dagger } {c_i} . \end{aligned}$$

12 $$\begin{aligned} H_{2}&= - t \sum _{i=1}^{N-1} ( {c_i}^{\dagger } {c_{i+1}} + h.c ) + \Delta \sum _{i=1}^{N-1} ( {c_i} {c_{i+1}} + h.c ) + U \sum _{i=1}^{N-1} ( 2 {c_i}^{\dagger } {c_i} - 1) ( 2 {c_{i+1}}^{\dagger } {c_{i+1}} - 1) -{\mu } \sum _{i}^{N} {c_i}^{\dagger } {c_i} . \end{aligned}$$

We recast this model Hamiltonian in terms of spin-1/2 operators by using the Jordan-Wigner transformation to connect the spinless fermion operators to the spin-1/2 operator, which is below.

$$ {c_j}^{\dagger } = ( {s_j}^{+}) {\Pi }_{l=1}^{j-1} ( {-s_l}^{z} ) $$. $$ {c_j} = ({s_j}^{-}) {\Pi }_{l=1}^{j-1} ( {-s_l}^{z} ) $$.

After this transformation the Kitaev Hamiltonian become

13 $$\begin{aligned} H_1 = \sum _n [(\frac{t+\Delta }{2})S_n^x S_{n+1}^x + \frac{(t-\Delta )}{2} S_n^y S_{n+1}^y - \mu S_n^z]. \end{aligned}$$

14 $$\begin{aligned} H_2 = \sum _n [(\frac{t+\Delta }{2})S_n^x S_{n+1}^x + \frac{(t-\Delta )}{2} S_n^y S_{n+1}^y + U S_n^z S_{n+1}^z - \mu S_n^z]. \end{aligned}$$

The above two Hamiltonians are free from *K*. Therefore, now our main task is to find the analytical expression for spin-1/2 operators in terms of in terms of bosonized fields $\phi$ and $\theta$ and that also show how *K* appears in the Kitaev model.

We present spin operators interms of $\phi$, $\theta$ and *K*^29,30^,

15 $$\begin{aligned} {S_n}^{x}&= [ \cos (2 \sqrt{\pi K} \phi (x) ) + {(-1)}^{n} ] \cos (\sqrt{\frac{\pi }{K}} \theta (x) , \end{aligned}$$

16 $$\begin{aligned} {S_n}^{y}&= - [ \cos (2 \sqrt{\pi K} \phi (x) ) + {(-1)}^{n} ] \sin (\sqrt{\frac{\pi }{K}} \theta (x) , \end{aligned}$$

17 $$\begin{aligned} {S_n}^{z}&= {(-1) }^{n} \cos (2 \sqrt{\pi K} \phi (x) ) + \sqrt{\frac{K}{\pi }} {\partial _x} \phi (x). \end{aligned}$$

Bosonized version of non-interacting and interacting Hamiltonians are the following.

18 $$\begin{aligned} H_1 = H_0 + \frac{\Delta }{2} \int \cos \left( 2 \sqrt{\frac{\pi }{K}}\theta (x)\right) dx - \mu \sqrt{\frac{K}{\pi }} \int (\partial _x \phi (x)) dx, \end{aligned}$$

19 $$\begin{aligned} H_2 = H_0 + \frac{\Delta }{2} \int \cos \left( 2 \sqrt{\frac{\pi }{K}}\theta (x)\right) dx + U \int \cos \left( 4 \sqrt{\pi K}\phi (x)\right) dx - \mu \sqrt{\frac{K}{\pi }} \int (\partial _x \phi (x)) dx, \end{aligned}$$

$H_0 = \frac{v}{2} \int [(\partial _x \theta )^2+ (\partial _x \phi )^2] dx$. We notice that $H_0$ appears with out *K*, therefore the rest three terms of Eq. (17) appear as a function of *K* otherwise it appears with out *K*.

### Derivation of exact solutions for the non-interacting RG equation (Eq. 3)

We may write the equation for $\frac{d \Delta }{dK}$ from Eq. (9) as

$$\begin{aligned} \frac{d \Delta }{d K} = \frac{ 8 [2 - \frac{1}{K} (1 + \frac{\mu }{\pi \sqrt{\pi }})]}{\Delta } \end{aligned}$$

We can write the integration constant as

$$\begin{aligned} C = {\frac{1}{2} {\Delta }^2 } -16 K + 8 (1 + \frac{\mu }{\sqrt{\pi }}) ln (K). \end{aligned}$$

One can evaluate the constant from the initial value of $$\Delta $$ and *K*, i.e., $${\Delta }_0 $$ and $$K_0. $$

$$\begin{aligned} \Delta =\sqrt{ { {\Delta _0}^2 } + 32 (K-K_0) - 16 (1 + \frac{\mu }{\pi \sqrt{\pi }} ) ln (K/{K_0}) }. \end{aligned}$$

Finally we obtain the exact solution for RG flow lines by integrating the RG equations.

### Derivation of renormalization group equations for non-interacting and interacting Hamiltonians

Our starting point is the bosonized Hamiltonian,

20 $$\begin{aligned} H = H_0 + a \int \cos \left( 2 \sqrt{\frac{\pi }{K}}\theta (x)\right) dx + \mu \sqrt{\frac{K}{\pi }} \int (\partial _x \phi (x)) dx, \end{aligned}$$

where $H_0 = \frac{v}{2} \int [(\partial _x \theta )^2+ (\partial _x \phi )^2] dx$. Here, we consider ${\Delta }=2a$ for the smoothness of calculation, but we finally present the RG equations in terms of $\Delta$. Now we write the partition function ${\mathscr {Z}}$ in terms of fields as,

21 $$\begin{aligned} {\mathscr{Z}}= \int {\mathscr {D}}\phi {\mathscr {D}}\theta e^{-S_E[\theta ]}, \end{aligned}$$

where $S_E$ is the Euclidean action which can be written as $S_E= -\int dr {\mathscr {L}} = -\int dr ({\mathscr {L}}_0 + {\mathscr {L}}_{int})$, where $r=(\tau ,x)$. Now we divide the fields into slow and fast modes and integrate out the fast modes. $\theta$ is $\theta (r)=\theta _s(r)+\theta _f(r)$, where

22 $$ \begin{aligned} \theta _s(r)=\int _{-\Lambda /b}^{\Lambda /b} \frac{d\omega }{2\pi } e^{-i\omega r}\theta (\omega ) \;\;\;\; \& \;\;\; \theta _f(r)=\int _{\Lambda /b<|\omega _n|<\Lambda } \frac{d\omega }{2\pi } e^{-i\omega r}\theta (\omega ). \end{aligned}$$

Here $\Lambda$ is the cut-off to start with and *b* is a factor greater than one. It is clear from the above definitions of faster and slower mode. One can make the average over the fast mode in order to get an effective action for the slower mode. Thus ${\mathscr {Z}}$ is

23 $$\begin{aligned} {\mathscr {Z}}=\int {\mathscr {D}}\theta _s {\mathscr {D}}\theta _f e^{-S_s(\phi _s,\theta _s)} e^{-S_f(\phi _f,\theta _f)} e^{-S_{int}(\phi ,\theta )}. \end{aligned}$$

Using the relation $\left\langle A \right\rangle _f = \int {\mathscr {D}}\theta _f e^{-S_f(\theta _f)} A$, one can write

24 $$\begin{aligned} {\mathscr {Z}}=\int {\mathscr {D}}\theta _s e^{-S_s(\theta _s)} \left\langle e^{-S_{int}(\theta )} \right\rangle _f. \end{aligned}$$

We write the effective action as

25 $$\begin{aligned} e^{-S_{eff}(\theta _s)}=e^{-S_s(\theta _s)} \left\langle e^{-S_{int}(\theta )} \right\rangle _f. \end{aligned}$$

Taking $\ln$ on both sides give, $S_{eff}(\theta _s)= S_s(\theta _s) - \ln \left\langle e^{-S_{int}(\theta )} \right\rangle _f.$ By writing the cumulant expansion up to third order, we have

26 $$\begin{aligned} S_{eff}(\theta _s) & = S_s(\theta _s)+ \left\langle S_{int}(\theta ) \right\rangle _f - \frac{1}{2} \left( \left\langle S^2_{int}(\theta ) \right\rangle _f - \left\langle S_{int}(\theta ) \right\rangle ^2_f \right) \\ & \quad +  \frac{1}{6} \left( \left\langle S^3_{int}(\theta ) \right\rangle _f - 3 \left\langle S^2_{int}(\theta ) \right\rangle _f \left\langle S_{int}(\theta ) \right\rangle _f + 2 \left\langle S_{int}(\theta ) \right\rangle ^3_f \right) . \end{aligned}$$

At first we derive the 2nd order RG equations for $\mu =0$ and then we extend it for finite $\mu$.

27 $$\begin{aligned} \left\langle S_{int}(\theta )\right\rangle = \int dr \left[ a\left\langle \cos \left( 2 \sqrt{\pi } \theta (r) \right) \right\rangle _f \right] \end{aligned}$$

28 $$\begin{aligned} \int dr \left[ a\left\langle \cos \left( 2 \sqrt{\pi } \theta (r)\right) \right\rangle _f \right] = b^{-\frac{1}{K}} \int dr \left[ a \cos \left( 2 \sqrt{\pi } \theta _s(r)\right) \right] \end{aligned}$$

Now we calculate the second order cumulant term of the action $S_{eff} (\theta )$ (Eq. 25).

29 $$\begin{aligned} -\frac{1}{2}(\left\langle S_{int}^2\right\rangle -\left\langle S_{int}\right\rangle ^2) = -\frac{1}{2} \int dr dr^{\prime } \left\{ a^2 [....] \right\} , \end{aligned}$$

where the dotted term represents the expectation value of the correlation function of sine-Gordon operators, which we evaluate below.

30 $$\begin{aligned}&-\frac{a^2}{2} \int dr dr^{\prime } \left\{ \left\langle \cos [2\sqrt{\pi }\theta (r)] \cos [2\sqrt{\pi }\theta (r^{\prime })] \right\rangle \right. \\&\quad \left. - \left\langle \cos [2\sqrt{\pi }\theta (r)] \right\rangle \left\langle \cos [2\sqrt{\pi }\theta (r^{\prime })] \right\rangle \right\} \\&\quad = \frac{a^2}{4} \left( 1-b^{-\frac{2}{K}} \right) \int dr (\partial _r \theta _s(r))^2. \end{aligned}$$

We obtain the following relation by comparison of rescaled *a* term using the rescaled relation as $b = e^{dl}$.

$$\begin{aligned} {\bar{a}}= a b^{(2-\frac{1}{K})} \end{aligned}$$

Finally we write the RG equation in terms of $\Delta (=2a)$ as,

$$\begin{aligned} \frac{d \Delta }{dl}= \left( 2-\frac{1}{K}\right) \Delta \end{aligned}$$

Comparison of rescaled *K* terms from the contribution of $\phi$, gives,

$$\begin{aligned} {\bar{K}}= K + \frac{Ka^2}{4}\left( b^2 - b^{(2-\frac{2}{K})}\right) \end{aligned}$$

We obtain the final form of $\frac{dK}{dl}$ after the contribution from $\theta$ for *K*.

$$\begin{aligned} \dfrac{dK}{dl}= \frac{\Delta ^2}{8} \end{aligned}$$

Similarly one can find the complete RG equation in the following way.

Now we calculate the third order terms of cumulant expansion for the effective action (Eq. 25),

$$\begin{aligned} \frac{1}{6}\left( \left\langle S_{int}^3 \right\rangle -3\left\langle S_{int}^2 \right\rangle \left\langle S_{int} \right\rangle +2 \left\langle S_{int} \right\rangle ^3\right) = \frac{1}{6} \int d\tau d\tau ^{\prime } d\tau ^{\prime \prime }\\ \left\{ a^3 \left[ ..... \right] + U^3 \left[ ..... \right] + a^2 U \left[ ..... \right] + a U^2 \left[ ..... \right] \right\} \end{aligned}$$

31 $$\begin{aligned}{}&\frac{a^3}{6} \int d\tau d\tau ^{\prime } d\tau ^{\prime \prime } \left[ \left\langle \cos [\sqrt{4\pi }\theta (\tau )] \cos [\sqrt{4\pi }\theta (\tau ^{\prime })] \cos [\sqrt{4\pi }\theta (\tau ^{\prime \prime })] \right\rangle \right. \\&\quad \left. - 3 \left\langle \cos [\sqrt{4\pi }\theta (\tau )] \cos [\sqrt{4\pi }\theta (\tau ^{\prime })] \right\rangle \left\langle \cos [\sqrt{4\pi }\theta (\tau ^{\prime \prime })] \right\rangle \right. \\&\quad \left. + 2 \left\langle \cos [\sqrt{4\pi }\theta (\tau )] \right\rangle \left\langle \cos [\sqrt{4\pi }\theta (\tau ^{\prime })] \right\rangle \left\langle \cos [\sqrt{4\pi }\theta (\tau ^{\prime \prime })] \right\rangle \right]  \\ & = \left( \frac{a^3}{24}\right) (6b^{-\frac{3}{K}}-3b^{-\frac{5}{K}}-3b^{-\frac{1}{K}}) \int d\tau \cos [\sqrt{4\pi }(\theta _s(\tau ))]. \end{aligned}$$

Following the same procedure used to derive the second order RG, we obtain the following equations.

$$\begin{aligned} da= \left( 2-\frac{1}{K}\right) a dl \end{aligned}$$

Finally we write this equation in term of $\Delta$,

$$\begin{aligned} \frac{d \Delta }{dl}= \left( 2-\frac{1}{K}\right) \Delta \end{aligned}$$

We obtain, by comparing the $\theta$ terms,

32 $$\begin{aligned} \frac{dK}{dl}= \frac{a^2}{2}= \frac{\Delta ^2 }{8} \end{aligned}$$

## Modified RG equations in presence of $\mu$

Now we derive the RG equations for finite chemical potential ($\mu \ne 0$). The presence of finite chemical potential yields five extra terms in the second order cumulant expansion (Eq. 29), of which three are vanishing. The non-vanishing contributions are from the two correlation functions, (a). $\int dr dr^{'} a i \frac{\mu }{\sqrt{\pi }}$, and (b). $\int dr dr^{'} i \frac{\mu }{\sqrt{\pi }} a$,

Now we calculate $a i \frac{\mu }{\sqrt{\pi }}$ term:

$$\begin{aligned}{}&-\frac{1}{2} \int d\tau d\tau ^{\prime }\left( - \frac{a i}{\mu } \sqrt{\pi } \left\langle \cos (\sqrt{4\pi }\theta (\tau )) \partial _{\tau ^{\prime }}\theta (\tau ^{\prime })\right\rangle - \left\langle \cos (\sqrt{4\pi }\theta (\tau )) \right\rangle \left\langle \partial _{\tau ^{\prime }}\theta (\tau ^{\prime })\right\rangle \right) \nonumber \\&\quad = \frac{a i \mu }{2 \sqrt{\pi }}\int d\tau d\tau ^{\prime } \left[ \left\langle \cos [\sqrt{4\pi }\theta (\tau )] (\partial _{\tau ^{\prime }}\theta _s(\tau ^{\prime }) +\partial _{\tau ^{\prime }}\theta _f(\tau ^{\prime }))\right\rangle \right. \nonumber \\&\qquad \left. - \left\langle \cos [\sqrt{4\pi }\theta (\tau )] \right\rangle \left\langle \partial _{\tau ^{\prime }}\theta _s(\tau ^{\prime }) +\partial _{\tau ^{\prime }}\theta _f(\tau ^{\prime }) \right\rangle \right] \nonumber \\&\quad =\frac{a i \mu }{2 \sqrt{\pi }}\int d\tau d\tau ^{\prime }\left[ \left\langle \cos [\sqrt{4\pi }\theta (\tau )]\partial _{\tau ^{\prime }} \theta _f(\tau ^{\prime })\right\rangle \right] \nonumber \\&\quad =-\frac{a \mu }{\sqrt{\pi } 2\pi } (1-b^{-\frac{1}{K}})\int d\tau \cos [\sqrt{4\pi }\theta _s(\tau )] \end{aligned}$$

Similarly one can calculate the, $\int dr dr^{'} \frac{i \mu }{ \sqrt{\pi }} a$.

Finally, combination of these two terms ($a+b$) yields $-\frac{a \mu }{\pi \sqrt{\pi }} (1-b^{-\frac{1}{K}})\int d\tau \cos [\sqrt{4\pi }\theta _s(\tau )$, as a consequence of it *a* term modify to

$$\begin{aligned} {\bar{a}} =a\left( b^{2-\frac{1}{K}}\right) -\frac{ a \mu }{ \pi \sqrt{\pi }} (b^2-b^{2-\frac{1}{K}}) . \end{aligned}$$

Finally we obtain the modified 2nd order and 3rd RG equations in presence of $\mu$ from the rescaled *a* term as

$$\begin{aligned} {\bar{a}} =a \left( b^{2-\frac{1}{K}}\right) - \frac{ a \mu }{\pi \sqrt{\pi }} (b^2-b^{2-\frac{1}{K}}) + \left( \frac{a^3}{24}\right) (6b^{2-\frac{3}{K}}-3b^{2-\frac{5}{K}}-3b^{2-\frac{1}{K}}) \end{aligned}$$

The correlation functions for finite $\mu$, only show up for the RG equations of $\frac{da}{dl}$ because only these two correlation functions give non vanishing contributions. The 3rd order RG equation in terms of $\Delta$ as

33 $$\begin{aligned} \frac{d \Delta }{dl}= \left[ 2-\frac{1}{K}\left( 1+ \frac{\mu }{\sqrt{\pi }}\right) \right] \Delta \end{aligned}$$
